# Cyanide Metabolism in Apple Embryos During Dormancy Breakage Is Modulated by NO or HCN Application

**DOI:** 10.3390/biology15191736

**Published:** 2026-10-01

**Authors:** Maciej Arkadiusz Piekarniak, Katarzyna Ciacka, Agnieszka Gniazdowska, Urszula Krasuska

**Affiliations:** Department of Botany and Plant Physiology, Institute of Biology, Warsaw University of Life Sciences, Nowoursynowska 159, 02-776 Warsaw, Poland; katarzyna_ciacka@sggw.edu.pl (K.C.); agnieszka_gniazdowska@sggw.edu.pl (A.G.); urszula_krasuska@sggw.edu.pl (U.K.)

**Keywords:** β-cyanoalanine synthase, β-glucosidase, hydrogen cyanide, *Malus domestica* Borkh., nitric oxide, rhodanese, seeds

## Abstract

Hydrogen cyanide (HCN) is an N-containing gasotransmitter with a regulatory function in plants and animals. Despite its toxicity, HCN acts as a signalling molecule in plants, influencing development, pathogen defence, and stress responses, with particular emphasis on biotic stress and seed dormancy/germination. Around 3000 species across > 130 families produce HCN from cyanogenic compounds. This study investigated how nitric oxide (NO) and HCN impact the activity of enzymes involved in HCN metabolism during dormancy breakage of apple (*Malus domestica* Borkh.) embryos. Based on earlier findings that HCN exposure elevates NO emission and alters the phytoglobin–NO cycle, we examined whether NO affects HCN metabolism. We measured the activities of β-glucosidases, β-cyanoalanine synthase (β-CAS), and sulfurtransferases (STRs) (HCN-detoxifying enzymes), and the transcript levels of their corresponding gene homologues. Both NO and HCN stimulated β-glucosidase activity and enhanced β-CAS and STR activities. Although both N-containing gasotransmitters increased β-CAS transcripts, they differentially implicated transcript levels of STR homologues. Additionally, only HCN increased the transcript levels of mitochondrial cysteine synthase (*OASTL-C*). These results, together with our previous work, potentially link NO and HCN in the metabolic network during dormancy breakage of apple embryos.

## Simple Summary

Although nitric oxide and hydrogen cyanide are toxic at high levels, plants produce them in small amounts as signalling molecules. During cold stratification of apple seeds (time duration: 40–90 days), which mimics the natural alleviation of dormancy, both gases are released. The short-term fumigation of dormant apple embryos with nitric oxide or hydrogen cyanide stimulates germination and seedling development. After exposure of apple embryos to hydrogen cyanide or nitric oxide, we observed increased activities of β-glucosidases and enzymes involved in hydrogen cyanide detoxification (β-cyanoalanine synthase and sulfurtransferases), as well as differential alteration of transcript levels of genes encoding hydrogen cyanide detoxification enzymes. Stimulating the activity of enzymes involved in hydrogen cyanide detoxification may help to maintain this gas’s intracellular concentration. An increase in β-glucosidase, sulfurtransferase and β-cyanoalanine synthase activities after nitric oxide application reveals the indirect involvement of this molecule in hydrogen cyanide metabolism, potentially linking these nitrogen-containing gasotransmitters in the metabolic network regulating seed germination.

## 1. Introduction

**Figure 1:**
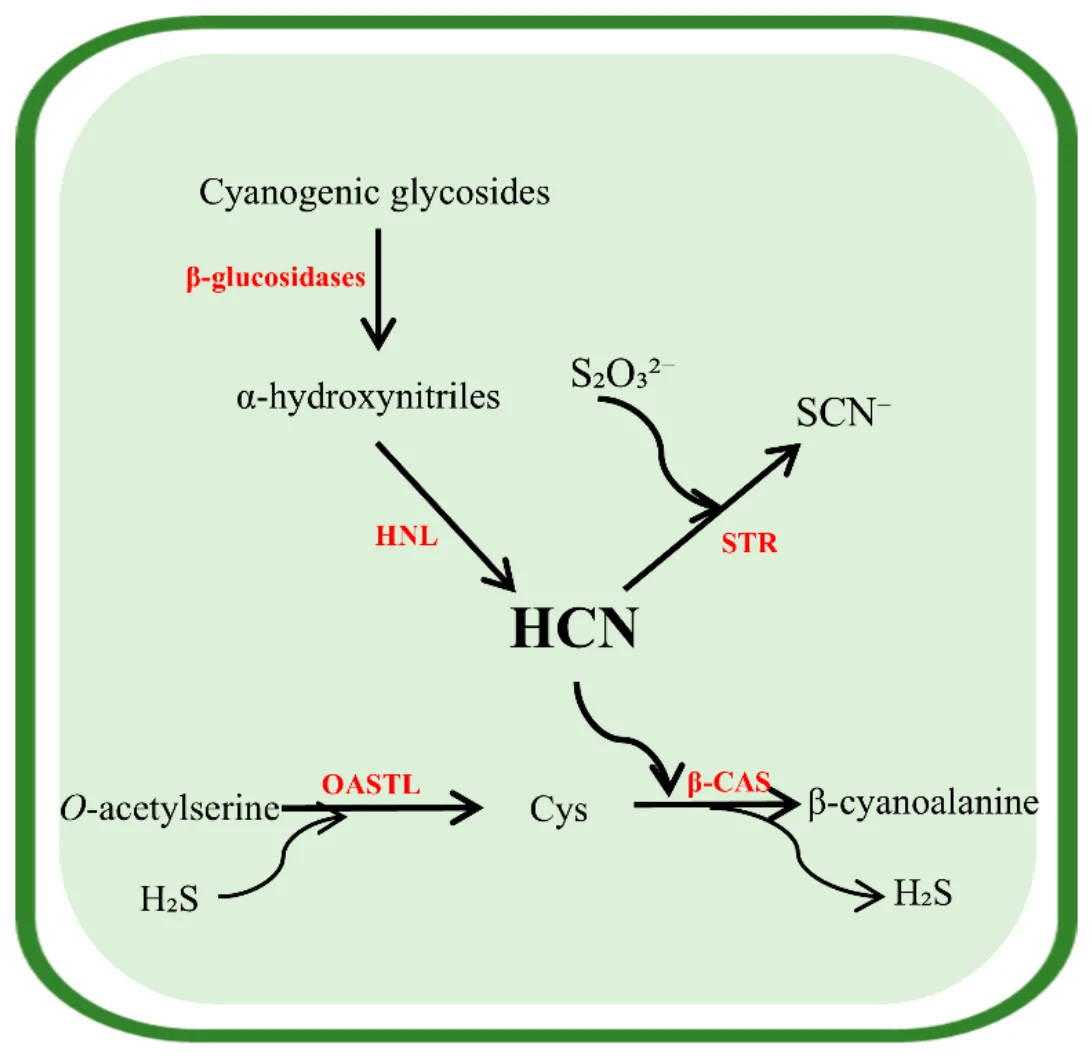
Schematic overview of the HCN liberation process and the main HCN detoxification pathway in plants. HCN release is accompanied by the breaking of β-glucosidic bonds in cyanogenic glycosides in the presence of β-glucosidases, forming α-hydroxynitriles, which are next metabolised by α-hydroxynitrile lyase (HNL). Cys participates in HCN detoxification via β-cyanoalanine synthase (β-CAS), which produces hydrogen sulfide (H_2_S). The last step in Cys synthesis (necessary for HCN detoxification) involves *O*-acetylserine (thiol) lyase (OASTL) isoforms, which catalyse the reaction between H_2_S (produced via the β-CAS pathway) and *O*-acetylserine. The alternative HCN detoxification pathway involves sulfurtransferases (STR) that possess rhodanese (Rhd) domains and use thiosulfate (S_2_O_3_^2−^) as a sulphur donor, producing thiocyanate (SCN^−^).

HCN is a member of the family of gasotransmitters, displaying a regulatory role in both plants and animals [1,2,3,4,5]. Although it has a “toxic nature”, HCN is increasingly recognisable as a signalling molecule in plants, playing a role in regulating various physiological processes [2]. The effect of the action of this N-containing gasotransmitter in plants has mainly been studied in relation to responses to biotic stressors [6,7,8,9,10] and regulation of seed dormancy and germination [11,12,13,14]. Approximately 3000 plant species from over 130 families can liberate HCN from stored cyanogenic compounds [15]. HCN is released mainly through (I) cyanogenic glycoside breakdown by β-glucosidase activity, (II) synthesis from 1-aminocyclopropane-1-carboxylic acid (ACC) during the ethylene biosynthetic pathway, (III) camalexin biosynthesis, (IV) synthesis from glyoxalate in the presence of hydroxylamine and from glycine [5,16,17,18,19]. HCN released from cyanogenic glycosides involves one or more β-glycosidases that cleave β-glycosidic bonds, producing an unstable cyanohydrin (α-hydroxynitrile) containing hydroxyl groups and a sugar moiety (Figure 1). This intermediate is rapidly degraded, either spontaneously or through enzymatic action by α-hydroxynitrile lyase (HNL). The breakdown of cyanohydrin yields HCN and a carbonyl compound, such as an aldehyde or ketone (Figure 1) [20,21,22]. Additionally, cyanide (CN^−^) can react with ketone groups in molecules and intermediates associated with Schiff bases, forming cyanohydrins and nitriles [23]. In apple seeds, the main cyanogenic glycosides are amygdalin, prunasin and neoamygdalin [24,25]. One of the primary functions of cyanogenic compounds is to serve as a defensive barrier against animal, microbial, and fungal attack. Cyanogenic glycosides are stored as inactive molecules and require enzymatic activation [26]. Herbivores disrupt plant tissue, allowing β-glucosidase to interact with cyanogenic glycosides, thereby cleaving the β-glucosidic bond and releasing α-hydroxynitriles (cyanohydrins) with defensive properties [27]. Thus, cyanogenic glycosides act as inactive precursors that generate a “defensive mechanism” against herbivores at the site and time of injury, controlled by enzyme and substrate compartmentalisation [7,28,29,30]. Besides their protective role against herbivores or pathogen attack, they also act as an alternative nitrogen source [31].

HCN detoxification is tightly regulated by two different enzymes: β-cyanoalanine synthase (β-CAS, EC 4.4.1.9) and sulfurtransferases (thiosulfate: cyanide sulfurtransferase; E.C. 2.8.1.1), also called rhodanese (Figure 1) [32].

Cysteine (Cys) serves as a precursor for various other sulphur-containing metabolites, including methionine, glutathione (GSH), and iron-sulphur (Fe-S) clusters, all of which play crucial roles in plant growth, development, and stress response [33,34,35,36]. Cys is also implicated as a substrate for β-CAS-mediated HCN assimilation in plants (Figure 1), highlighting the tight metabolic interplay between hydrogen sulphide (H_2_S) and Cys turnover, particularly under stress conditions [37]. In plant cells, Cys biosynthesis occurs across multiple compartments, including cytosol, mitochondria, and chloroplasts [38]. This process is accompanied by Cys synthase (CS), a multi-protein complex, where *O*-acetylserine(thiol)lyase (OASTL; EC 4.2.99.8) is one of the components that catalyses the final step in Cys biosynthesis by combining *O*-acetylserine (OAS) with H_2_S (Figure 1) [39,40]. The CS gene family was first characterised in Arabidopsis, where it comprises 9 members grouped into 5 subfamilies; all of CS proteins possess a conserved pyridoxal 5′-phosphate (PLP) binding domain [41]. Besides these, OASTL-A1 (cytosolic), OASTL-B (plastidial), and OASTL-C (mitochondrial) isoforms exhibit higher expression levels compared to other family members [38,42,43]. In Arabidopsis chloroplasts, two OASTL homologues are present, encoded by the *OASTL-B* and *CS26* genes. Biochemical characterisation of recombinant CS26 revealed that this protein functions as an *S*-sulfocysteine synthase rather than a typical *O*-acetylserine(thiol)lyase [44]. Furthermore, the Arabidopsis CS family member CYS-C1 exhibits β-CAS activity and contributes to HCN detoxification (Figure 1). CYS-D1 and CYS-D2, localised in the cytoplasm, exhibit relatively low expression levels and are considered less active CSs [41]. β-CAS is a PLP-dependent enzyme, present in all plant organs, and localised in mitochondria. β-CAS is involved in regulating HCN homeostasis as well as in nitrogen and sulphur metabolism in both cyanogenic (containing cyanogenic glycosides) and non-cyanogenic plants [45]. This enzyme’s mode of action involves replacing the Cys sulfhydryl group with the CN^−^, forming β-cyanoalanine. During this reaction, H_2_S is produced (Figure 1) [6,45].

Sulfurtransferases (STRs, EC 2.8.1.x) are widespread enzymes found in bacteria, archaea and eukaryotes, and constitute a large and complex protein family characterised by the presence of a rhodanese (Rhd) domain and implicated in diverse molecular and signalling processes as sulphur carriers [46]. The STRs with a single Rhd domain include the so-called thiosulfate-STRs (TSTs), formerly rhodanese, based on their ability to use thiosulfate (S_2_O_3_^2−^) as a sulphur donor or to detoxify HCN by forming thiocyanate (SCN^−^) in in vitro assays (Figure 1) [46]. The identification of two cDNAs encoding rhodanese isoforms in Arabidopsis provided strong evidence for their presence in plants [47]. Moreover, single Rhd domain-containing STRs include cell division cycle 25 (Cdc25) phosphatases, which play an important role in cell cycle regulation [48]. A second major class comprises STRs with two Rhd domains, commonly present across diverse organisms and known as 3-mercaptopyruvate (3-MP)-STRs (MSTs) (EC 2.8.1.2) due to their capacity to utilise 3-MP as a substrate in vitro [46]. However, not all proteins with two Rdh domains exhibit MST activity. Finally, another group comprises STRs that contain an Rhd domain with a catalytic Cys fused to a domain with another function, thereby conferring specific roles [46]. In Arabidopsis, 20 proteins containing the Rhd domain have been identified. These were initially designated STR1-STR18 [49], with STR4a and STR17a later included following additional analyses [50]. They are divided into 6 subgroups [49]: I (STR1 and STR2), II (STR3, 4, and 4a), III (STR5-8), IV (STR9-11), V (STR12 and STR13) and VI (STR14-18) based on sequence homology [51]. Besides the role of STRs in CN^−^ detoxification [52], their involvement in sulphate assimilation [53] and in the mobilisation of sulphur for Fe-S cluster biosynthesis or repair [54] was also indicated.

Apple (*Malus domestica* Borkh.) seeds, classified as *orthodox* type, are plant material in studies focused on the physiology of oily, perennial, dicotyledonous seeds [25] and in the role of gasotransmitters in the regulation of seed dormancy breakage and germination [1,55,56]. Dormancy of apple seeds can be broken after 40–90 days of cold stratification at 5 °C [25,56,57,58]. HCN emission has been detected from apple seeds undergoing cold stratification [57] as well as from isolated dormant embryos whose culture was preceded by HCN short-term pre-treatment [59]. In apple seeds, under natural conditions typical of temperate zones, HCN released from cyanogenic glycosides (e.g., amygdalin) in apple seeds due to the activity of β-glucosidase occurs at chilling temperatures (above 0 °C) [25]. Low temperatures are crucial for this process, and the physiological effects of cold stratification can be mimicked by short-term HCN fumigation of isolated embryos. After such treatment, embryo dormancy is broken, and undisturbed seedling development is observed (approximately 7 days later). The peak concentration of free HCN in apple embryos occurs approximately on the 15th day of seed cold stratification [57]. Nitric oxide (NO) emission during cold stratification of apple seeds has also been identified; its peak concentration was detected on the 14th and 90th day of this process [60]. The interaction of these compounds in plants seems obvious. Whether NO can alter HCN-specific metabolic pathways remains unknown. Hence, the investigation of the roles of both N-containing gasotransmitters (NO and HCN) is needed for better understanding of the mechanisms underlying dormancy breakage. Short-term pre-treatment of dormant apple embryos with NO (for 3 h) or HCN (for 5 h) results in dormancy breaking, and seedling development after such pre-treatments appears more vigorous and shows no morphological abnormalities, as observed after seeds’ cold stratification [1,61]. Seed dormancy breakage and stimulation of germination after NO and HCN fumigation have also been demonstrated in many other plant species [62,63,64,65].

The aim of this study was to investigate the similarities in the action of NO and HCN, focusing on the pathways involved in HCN metabolism during dormancy breakage in apple embryos. In previous work, we observed elevated NO emissions following apple embryo exposure to HCN and alterations in elements of the phytoglobin-NO cycle that control intracellular NO levels, suggesting that HCN is implicated in NO metabolism [1,13]. In this work, we examine the role of NO exposition of apple embryos on the activity of β-glucosidases and enzymes implicated in HCN detoxification. The combination of fumigating apple embryos with HCN or NO will allow comparison of the actions of these two N-containing gasotransmitters when applied as a short pre-treatment at a high, non-physiological concentration. Use of HCN treatment will allow us to compare its effect with NO exposure and help to indicate whether NO can also affect HCN metabolism in apple embryos during dormancy breakage. To achieve the goal, we aimed to identify changes in the activities of β-glucosidases, β-CAS, and STRs, and in the transcript levels of OASTL, β-CAS, and STRs homologues during NO- or HCN-induced dormancy breakage in apple embryos.

## 2. Materials and Methods

### 2.1. Plant Material and Experimental Model

Plant material consisted of apple (*Malus domestica* Borkh., cv. Antonówka) seeds obtained from fruits produced by the local orchard WIKPOL (Grójec, Poland) in the 2025 season. After isolation and drying at room temperature, seeds were stored at 5 °C until use. Experiments were conducted on embryonic axes isolated from embryos fumigated with NO and HCN, as described in Piekarniak et al. [1].

Prior to the treatment, seeds were imbibed in water for 24 h. Embryos were then excised (Control, 0 h), and a portion was subjected to short-term fumigation with either (I) NO (3 h), generated from 20 mM sodium nitrite (NaNO_2_) acidified with 0.2 M HCl, or (II) HCN (5 h), released from 70 mM potassium cyanide (KCN) acidified with 0.2 M HCl, according to Gniazdowska et al. [61].

Isolated embryos were placed on moistened filter paper saturated with 0.01 M potassium phosphate buffer (pH 7.0) inside a 500 mL glass container. Within the container, separate beakers held acidified solutions of NaNO_2_ and KCN to generate NO and HCN, respectively. The container was tightly sealed for the entire treatment period. The exposure time and concentration of the gas-releasing solutions were pre-optimised for the current seed lot to reliably induce germination. Control embryos were incubated under identical conditions in sealed glass containers without any NO- or HCN-releasing solutions. Fumigation was carried out under the fume hood. Following fumigation, embryos were rinsed with distilled water, and the activated NO- or HCN-releasing solution was disposed of and utilised. Embryonic axes were immediately isolated from the part of the treated material, while the remaining embryos were placed on a moist filter in Petri dishes and cultured for 24 h in a growth chamber (Sanyo MLR-35OH, Osaka, Japan) under controlled conditions (25/20 °C day/night, 12/12 h photoperiod, light intensity of 100 µmol PAR m^−2^ s^−1^). After incubation, embryonic axes were collected (NO_24_ and HCN_24_). Axes isolated from non-treated embryos at 0, 3, 5, and 24 h served as control (C_0_, C_3_, C_5_, C_24_, respectively) (Figure S1). Embryonic axes of the apple embryos for all experiments were pooled from one Petri dish (approximately 25 axes for β-glucosidase, 50 axes for β-CAS and sulfurtransferase or 45 axes for transcript level analysis, randomly selected) to constitute one biological replicate for each time point and treatment, then frozen in liquid nitrogen and stored at −80 °C for further use.

### 2.2. Enzyme Purification and Activity Measurements

Embryonic axes (25 mg) for the extraction of β-glucosidase or embryonic axes (50 mg) for the extraction of β-CAS and STR were homogenised in liquid nitrogen; the obtained powder was transferred to Eppendorf tubes, and proteins were extracted using 300 µL of 50 mM potassium phosphate, pH 5.0 (β-glucosidase) or 400 µL 20 mM Tris-HCl, pH 8.0 (STR). After centrifugation, the endogenous CNˉ and SCN^−^ were removed using a concentrator PES, 3K MWCO (Thermo Scientific™, Waltham, MA, USA), at 15,000 *g* for 20 min at 4 °C. The protein concentration of the supernatant was adjusted to 5 µg µl^−1^ to ensure equal protein amounts across assay samples. Protein concentration was measured using a Bradford reagent [66] at 595 nm, with a microplate reader (ClarioStar Plus, BMG LABTECH, Ortenberg, Germany). Bovine serum albumin (BSA, Sigma-Aldrich, A7030, St. Louis, MO, USA) was used as a standard for protein quantification. All experiments were conducted with three biological replications and three technical repetitions (portions of the extract obtained from the axes of a single biological replicate) each.

#### 2.2.1. β-Glucosidase Activity Measurement

β-glucosidase activity was measured as described by Lecas et al. [67], with some modifications. Diluted supernatant (50 µL, 25 µg prot.) was mixed with 250 µL of 50 mM potassium phosphate, pH 5.0, and 5 mM *p*-nitrophenyl-β-D-glucopyranoside (*p*NPG; 35109, Cayman Chemical, Ann Arbor, Michigan, USA) in wells of a 96-well plate. The reaction was carried out for 30 min at 37 °C and stopped by adding 5 µL of 1 M sodium carbonate. β-glucosidase activity was measured at 405 nm using a microplate reader (ClarioStar Plus). The activity was calculated using the extinction coefficient for *p*-nitrophenol (*p*-NP, ε = 18,000 M^−1^ cm^−1^) and expressed as µmol *p*-NP mg prot.^−1^ min^−1^.

#### 2.2.2. β-CAS Activity Measurement

The β-CAS activity assay was based on Blumenthal et al. [68], with some modifications. The enzyme assay contained 5 mM L-Cys (C7352, Sigma-Aldrich, St. Louis, MO, USA), 15 mM KCN, 100 mM Tris-HCl (pH 9.0), and 100 µL enzyme extract (500 µg prot.), with a total volume of 500 µL. The reaction was initiated by the addition of L-Cys. After incubation for 30 min at 30 °C, the reaction was terminated by adding 500 µL of stop solution: 3 mM FeCl_3_ and 15 mM N, N’-dimethyl-*p*-phenylene-diamine (193992, Sigma-Aldrich, St. Louis, MO, USA) dissolved in 8.4 M HCl. The samples were then centrifuged at 13,000 *g* for 5 min to remove precipitated proteins. The formation of methylene blue was determined at 670 nm using a microplate reader (ClarioStar Plus). Quantification of enzymatically produced H_2_S was performed using solutions of different Na_2_S concentrations, treated in the same manner as the assay samples. The activity of β-CAS was expressed as pmol H_2_S mg prot^−1^ min^−1^.

#### 2.2.3. Rhodanese Activity Measurement

STR (thiosulfate cyanide sulfurtransferase) activity was estimated as described by Sörbo [69] with modifications by Papenbrock and Schmidt [70]. The assay mixture consisted of 100 mM Tris-HCl, pH 9.0, with 10 mM KCN, 5 mM mercaptoethanol, and 100 µL enzyme extract (500 µg prot.), and was initiated by adding 5 mM sodium thiosulfate (Na_2_S_2_O_3_). After incubation at 37 °C for 40 min, the reaction was stopped by the addition of 200 µL acidic iron reagent (FeCl_3_, 50 g L^−1^; 65% HNO_3_). After centrifugation at 13,000 *g* for 3 min, absorbance was measured at 460 nm using a microplate reader (ClarioStar Plus). The produced SCN^−^ from STR activity reacts with acidic iron reagent, forming red iron thiocyanate (Fe(SCN)_3_) complex. Product formation was quantified using a standard curve prepared with sodium thiocyanate (NaSCN). STR activity was expressed as pmol SCN^−^ mg prot.^−1^ min^−1^.

### 2.3. Transcript Level Analysis

Transcript level of mRNA encoding OASTL, β-CAS and STRs was performed in the embryonic axes by the qRT-PCR technique. Total RNA was isolated from 45 mg of embryonic axes using RNAzol^®^ RT (R4533, Sigma-Aldrich, Darmstadt, Germany), according to the manufacturer’s instructions. Residual genomic DNA was removed by treatment with DNase I (EN0521, Thermo Scientific™, Waltham, MA, USA). RNA concentration and purity were determined using a spectrophotometer (NanoDrop^TM^ 2000, Thermo Scientific™, Waltham, MA, USA). To synthesise cDNA, 200 ng of total RNA and RevertAid First Strand cDNA Synthesis Kit (Thermo Scientific #K1622) in a total volume of 10 μL was used. The product obtained was diluted 7-fold. The qRT-PCR was conducted on the Bio-Rad CFX Connect™ Real-Time PCR Detection System (Bio-Rad, Hercules, CA, USA) using iTaq™ universal SYBR^®^ Green supermix (Bio-Rad, Hercules, CA, USA). Reaction mixture containing 6 μL PCR supermix, 2 μL cDNA template, 1 μL primer and 3 μL H_2_O. Primers for transcript level measurements were designed using nucleotide sequences available in the National Center of Biotechnological Information (NCBI) database and the Genome Database for Rosaceae (GDR) (https://www.rosaceae.org/ (accessed on 5 May 2026)). Primer sequences, NCBI or GDR accession numbers, amplicon sizes, and PCR amplification efficiencies are provided in Table S1. Sequences were selected from Arabidopsis genes that are expressed in seeds, using the TAIR database. Gene nomenclature was defined based on similarity to Arabidopsis gene sequences.

PCR amplification efficiencies were determined using LinRegPCR software (v. 2021.2) by linear regression analysis of the log-linear phase of individual amplification curves, as described by Ruijter et al. [71]. The stability of five candidate reference genes was evaluated using NormFinder (v. 0.953) across all experimental conditions. *ACT* (encoding actin; Table S1) and *SAR1* (encoding a small GTP-binding protein; Table S1) genes were used as housekeeping genes to normalise transcript levels.

Normalised relative quantities were calculated using an efficiency-corrected relative quantification approach with normalisation based on the geometric mean of the two reference genes [72].

### 2.4. Statistics

Statistica13 Software (StatSoft Inc., Tulsa, OK, USA) was used for statistical analyses. The number of biological replicates (n) for each experiment is specified in the relevant figure legends. Differences among groups were evaluated after two-way analysis of variance (ANOVA), with type of gasotransmitter (NO or HCN) and exposure time as fixed factors, followed by homogeneous groups analysed using Tukey’s HSD test at *p* ≤ 0.05 for enzyme activities and log_2_-transformed normalised relative quantities. Prior to ANOVA, normality of variances was verified using the Shapiro–Wilk test. Corresponding *p*-values are reported in Table S2.

## 3. Results

### 3.1. NO and HCN Stimulate β-Glucosidase Activity in Embryonic Axes

**Figure 2:**
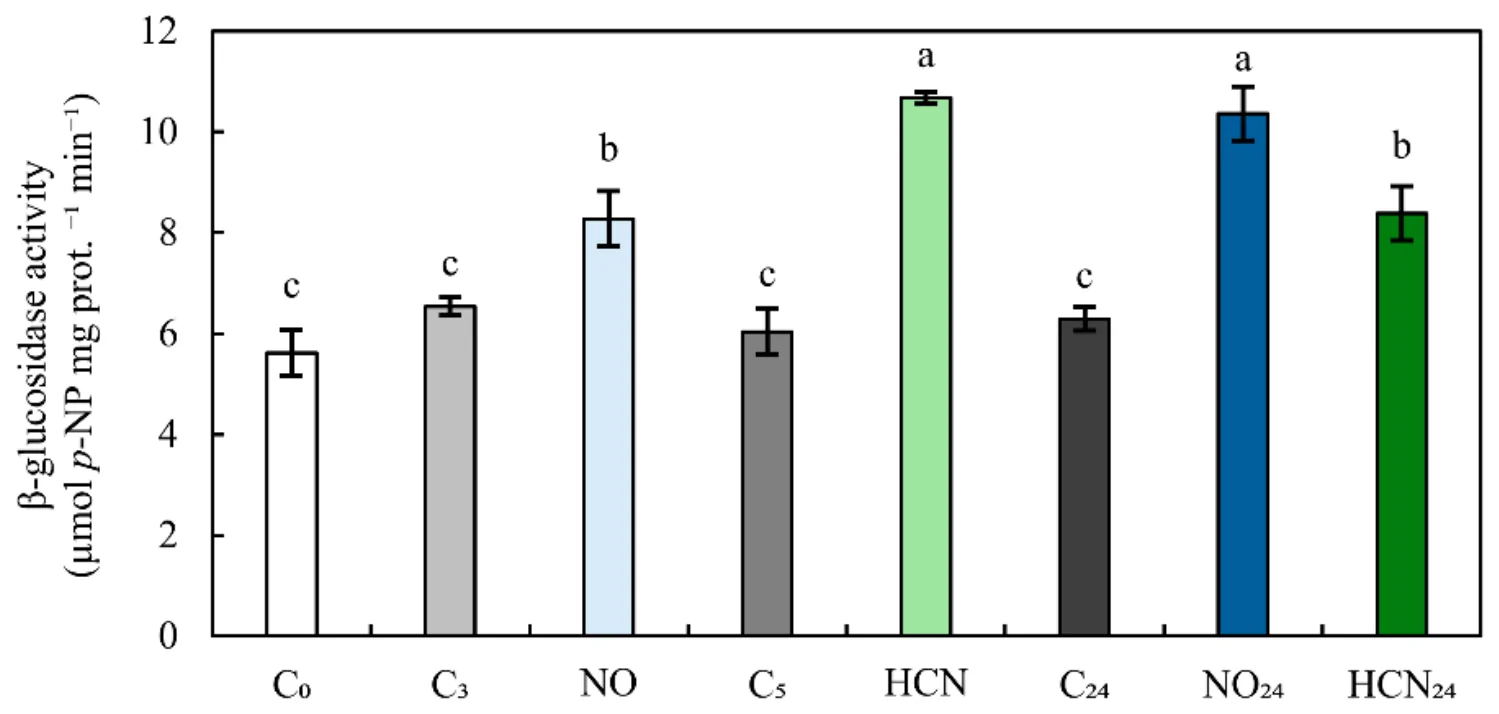
β-glucosidase activity in embryonic axes isolated from embryos not treated (just after isolation, C_0_), after culture in water for 3 h (C_3_), 5 h (C_5_) and 24 h (C_24_), and from embryos fumigated with NO or HCN, and after 24 h of post-fumigation culture in water (NO_24_, HCN_24_). Values are average ± SD, *n* = 3. Letters (a–c) indicate homogeneous groups identified according to Tukey’s post hoc test (*p* ≤ 0.05).

β-Glucosidase activity, expressed as the formation of *p*-NP after *p*NPG enzymatic decomposition, in the axes of non-treated apple embryos was stable throughout the experiment, at around 6 nmol *p*-NP mg prot.^−1^ min^−1^. NO application increased β-glucosidase activity both immediately after the treatment and after 24 h of prolonged culture (Figure 2). HCN exposure initially increased β-glucosidase activity, but as the culture progressed, the enzyme activity decreased significantly and remained higher than that in control axes (Figure 2).

### 3.2. β-CAS Activity in Embryonic Axes Is Elevated After NO and HCN Exposure

**Figure 3:**
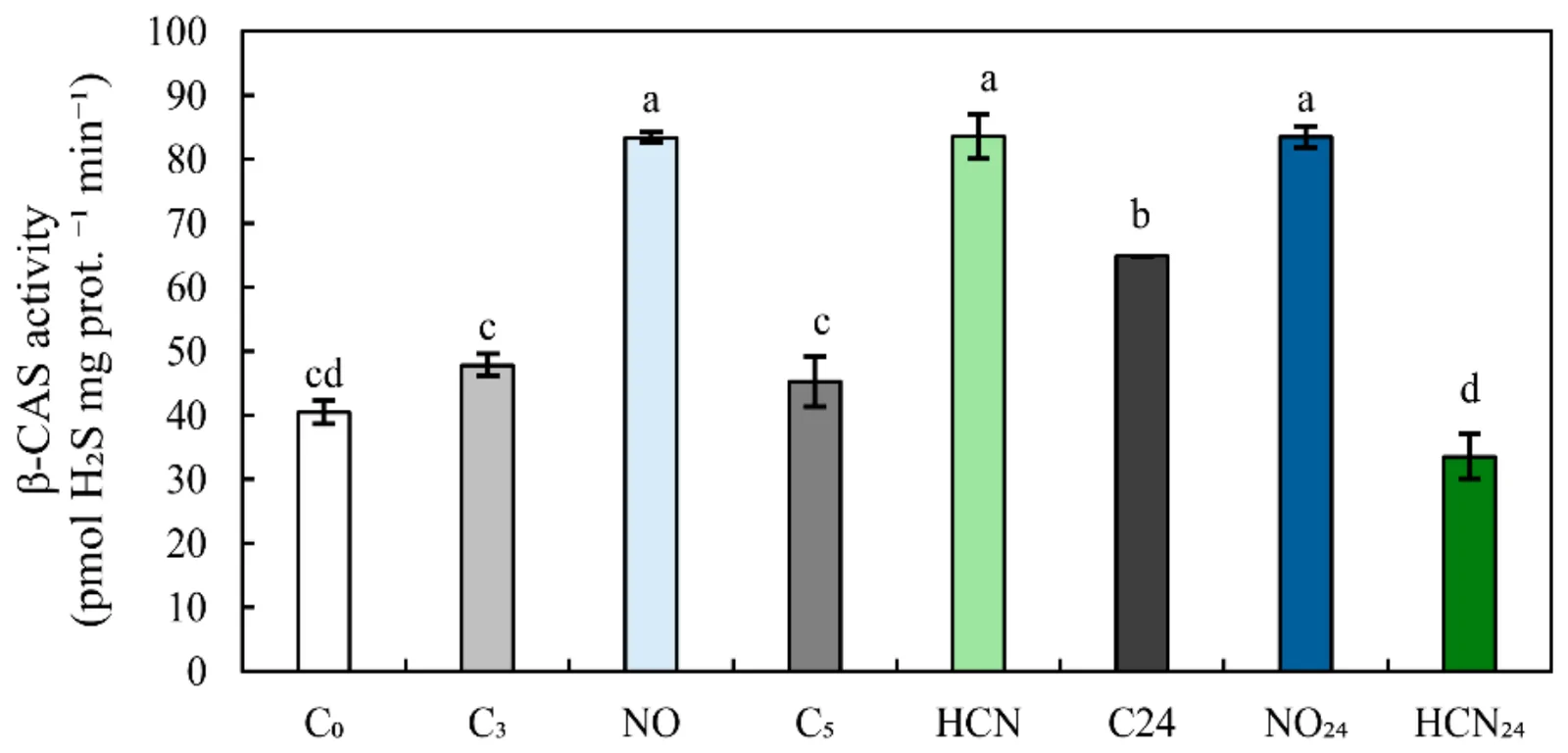
β-CAS activity in embryonic axes isolated from embryos not treated (just after isolation, C_0_), after culture in water for 3 h (C_3_), 5 h (C_5_) and 24 h (C_24_), and from embryos fumigated with NO or HCN, and after 24 h of post-fumigation culture in water (NO_24_, HCN_24_). Values are average ± SD, *n* = 3. Letters (a–d) indicate homogeneous groups identified according to Tukey’s post hoc test (*p* ≤ 0.05).

β-CAS activity was stimulated in apple embryonic axes just after NO and HCN treatment and after 24 h of NO post-fumigation culture (Figure 3). After HCN exposure, as the culture was prolonged for 24 h, its activity decreased in relation to axes isolated from non-treated embryos cultured for 24 h in water (C_24_) and to those after 5 h of HCN application (Figure 3). In axes from non-treated apple embryos, β-CAS activity remained stable during the initial period until 24 h, when it increased to a level 40% higher than that at the beginning of the experiment (Figure 3).

### 3.3. NO Increases Transcript Level of CYS-C1(β-CAS) and Has No Impact on CYS-D1, While HCN Exhibits Time-Dependent Increase in Transcript Levels of Both CYS-C1 (β-CAS) and CYS-D1

**Figure 4:**
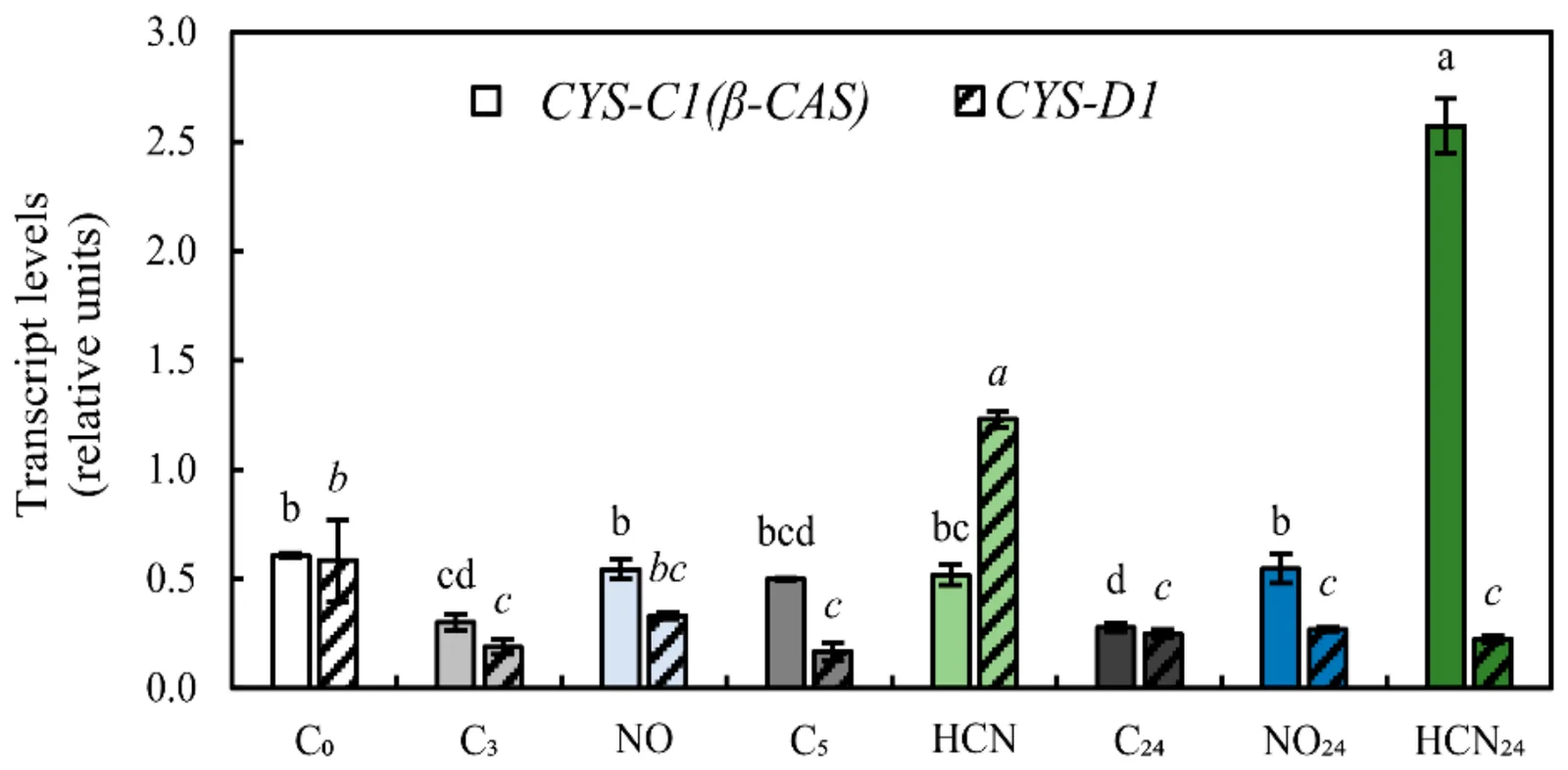
Transcript levels of *CYS-C1(β-CAS)* and *CYS-D1* isoforms of CS in embryonic axes isolated from embryos not treated (just after isolation, C_0_), after culture in water for: 3 h (C_3_), 5 h (C_5_) and 24 h (C_24_) and from embryos fumigated with NO or HCN, and after 24 h of post-fumigation culture in water (NO_24_, HCN_24_). Values are average ± SD, *n* = 3. For the *CYS-C1(β-CAS)* transcript level, letters (a–d) and *CYS-D1*, italics (*a*–*c*), indicate significant differences between the experimental combinations according to Tukey’s post hoc test (*p* ≤ 0.05).

Transcript levels of *CYS-C1(β-CAS)* and *CYS-D1* in embryonic axes from non-treated apple embryos lowered as culture was prolonged (Figure 4). NO application increased the transcript level of *CYS-C1(β-CAS)* after 3 h of treatment and after 24 h of water culture to the level observed in C_0_, whereas HCN exposure increased *CYS-C1(β-CAS)* transcript level only as the culture was prolonged to 24 h (Figure 4). The transcript level of *CYS-D1* was elevated in axes only just after HCN application (Figure 4).

### 3.4. OASTL-A1, OASTL-C and CS26 Transcript Levels Increase After HCN Exposure, Although Transcript Levels of Only CS26 Are Elevated in a Time-Dependent Manner by NO

**Figure 5:**
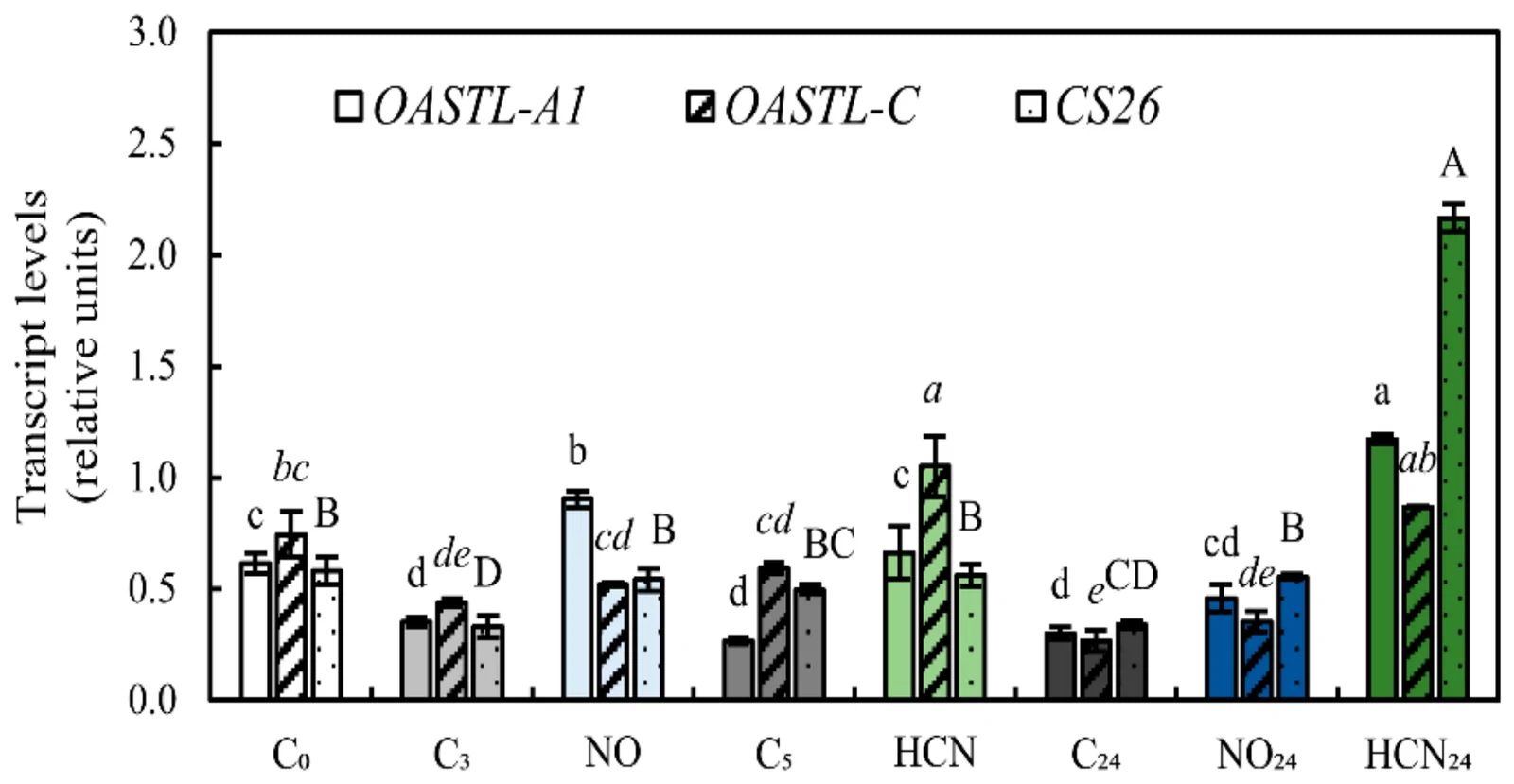
Transcript levels of CS (*OASTL-A1*, *OASTL-C* and *CS26*) homologues in axes of embryos isolated immediately after seed coat removal (C_0_) and after 3 h (C_3_), 5 h (C_5_), and 24 h (C_24_) of embryo culture, as well as in axes of embryos just after fumigation with NO or HCN (NO, HCN) and after 24 h of post-fumigation culture (NO_24_, HCN_24_). Values are average ± SD, *n* = 3. For the *OASTL-A1* transcript level, letters (a–d), *OASTL-C* transcript level, italics (*a*–*e*) and *CS26* transcript level, uppercase letters (A–D), indicate significant differences between the experimental combinations according to Tukey’s post hoc test (*p* ≤ 0.05).

In embryonic axes from non-treated apple embryos, *OASTL-A1*, *OASTL-C* and *CS26* transcript levels decreased slightly as the culture was prolonged for 24 h (Figure 5). Consistently, HCN treatment increased transcript levels of *OASTL-A1* and *OASTL-C* immediately after application and at 24 h post-fumigation culture, whereas *CS26* increased them only as the culture progressed (Figure 5). In contrast, NO increased *OASTL-A1* (only just after the treatment) and *CS26* transcript levels (after 3 h and 24 h), with no effect on the *OASTL-C* transcript level (Figure 5).

### 3.5. NO and HCN Treatment Stimulate STR Activity in Embryonic Axes

**Figure 6:**
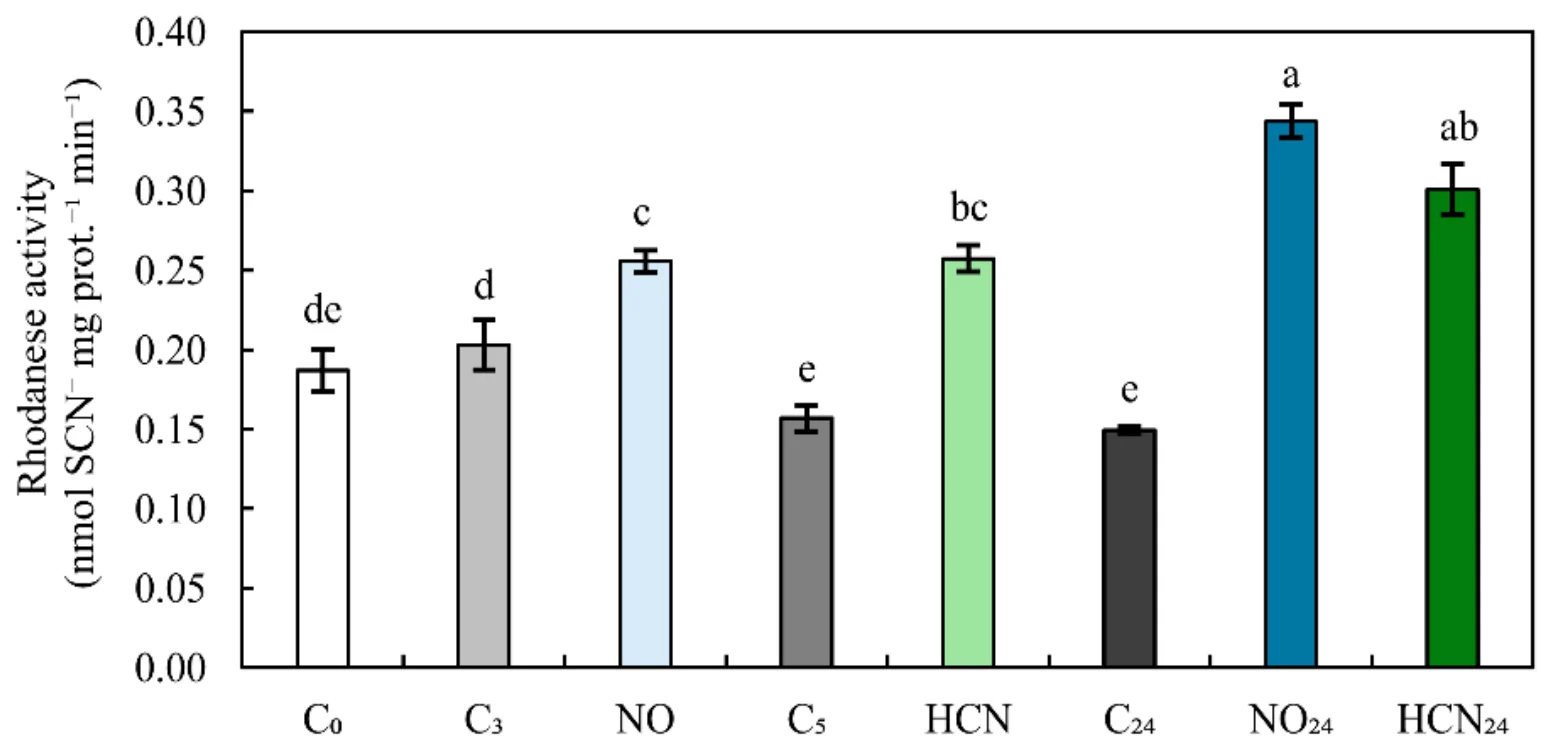
STR activity in axes of embryos isolated immediately after seed coat removal (C_0_) and after 3 h (C_3_), 5 h (C_5_), and 24 h (C_24_) of embryo culture, as well as in axes of embryos just after fumigation with NO or HCN (NO, HCN) and after 24 h of post-fumigation culture (NO_24_, HCN_24_). Values are average ± SD, *n* = 3. Letters (a–e) indicate homogeneous groups identified according to Tukey’s post hoc test (*p* ≤ 0.05).

STR activity was the highest after 24 h in post-fumigation culture after NO and HCN exposure (approx. 0.35 nmol SCN^−^ mg prot.^−1^ min^−1^) (Figure 6). NO and HCN also increased STR activity in embryonic axes, just after fumigation, as compared to the appropriate control (Figure 5), but not as strongly as after 24 h. In axes from non-treated embryos, STR activity increased slightly only in the short term after 3 h of imbibition, then decreased slightly as the culture was prolonged, reaching a stable value (Figure 6).

### 3.6. Only HCN Increases STR1, STR4, and STR4a Transcript Levels in Apple Embryonic Axes

**Figure 7:**
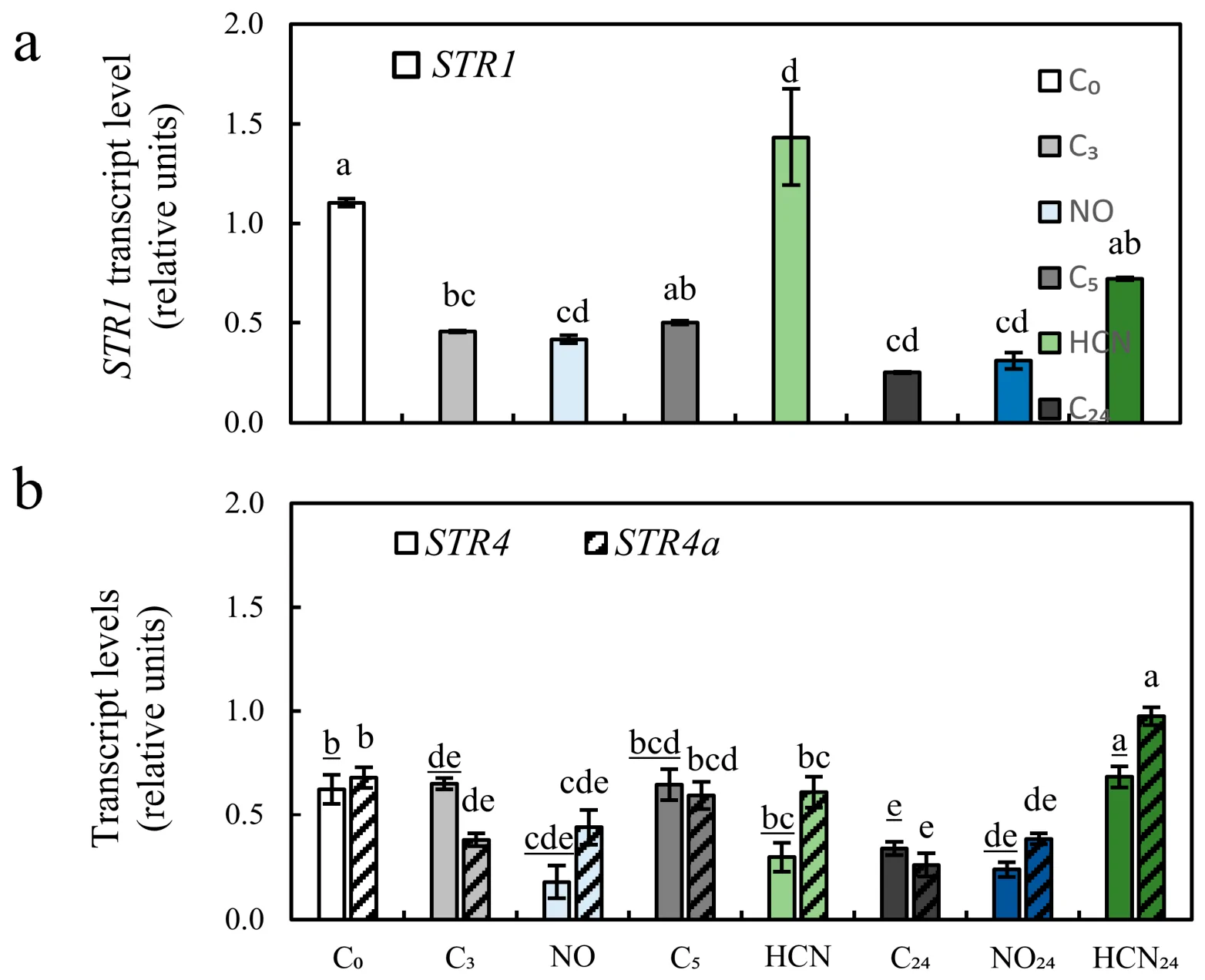
Transcript levels of (**a**) *STR1* and (**b**) *STR4* and *STR4a* in axes isolated from non-treated embryos (just after isolation, C_0_), after 3 h (C_3_), 5 h (C_5_) and 24 h (C_24_) imbibition in water and in axes of embryos just after their fumigation with NO (NO), or HCN (HCN) and after 24 h of post-fumigation culture (NO_24_, HCN_24_). Values are average ± SD, *n* = 3. For *STR1* transcript level, letters (a–d), *STR4* transcript level, underline letters (a–e), and *STR4a* transcript level, letters (a–e), indicate significant differences between the experimental combinations according to Tukey’s post hoc test (*p* ≤ 0.05).

HCN treatment of apple embryonic axes increased transcript levels of *STR1* (Figure 7a), as well as *STR4* and *STR4a*, but only after 24 h of post-fumigation culture (Figure 7b). NO application did not change transcript levels of the analysed genes (Figure 7a,b). In control embryonic axes isolated from non-treated embryos, transcript levels of all three genes decreased with prolonged culture duration (Figure 7a,b).

### 3.7. HCN Increases the Transcript Levels of STR6, STR7, STR8, STR9 and STR11, While NO Only Slightly Alters Transcript Levels of the Analysed Genes in Apple Embryonic Axes

**Figure 8:**
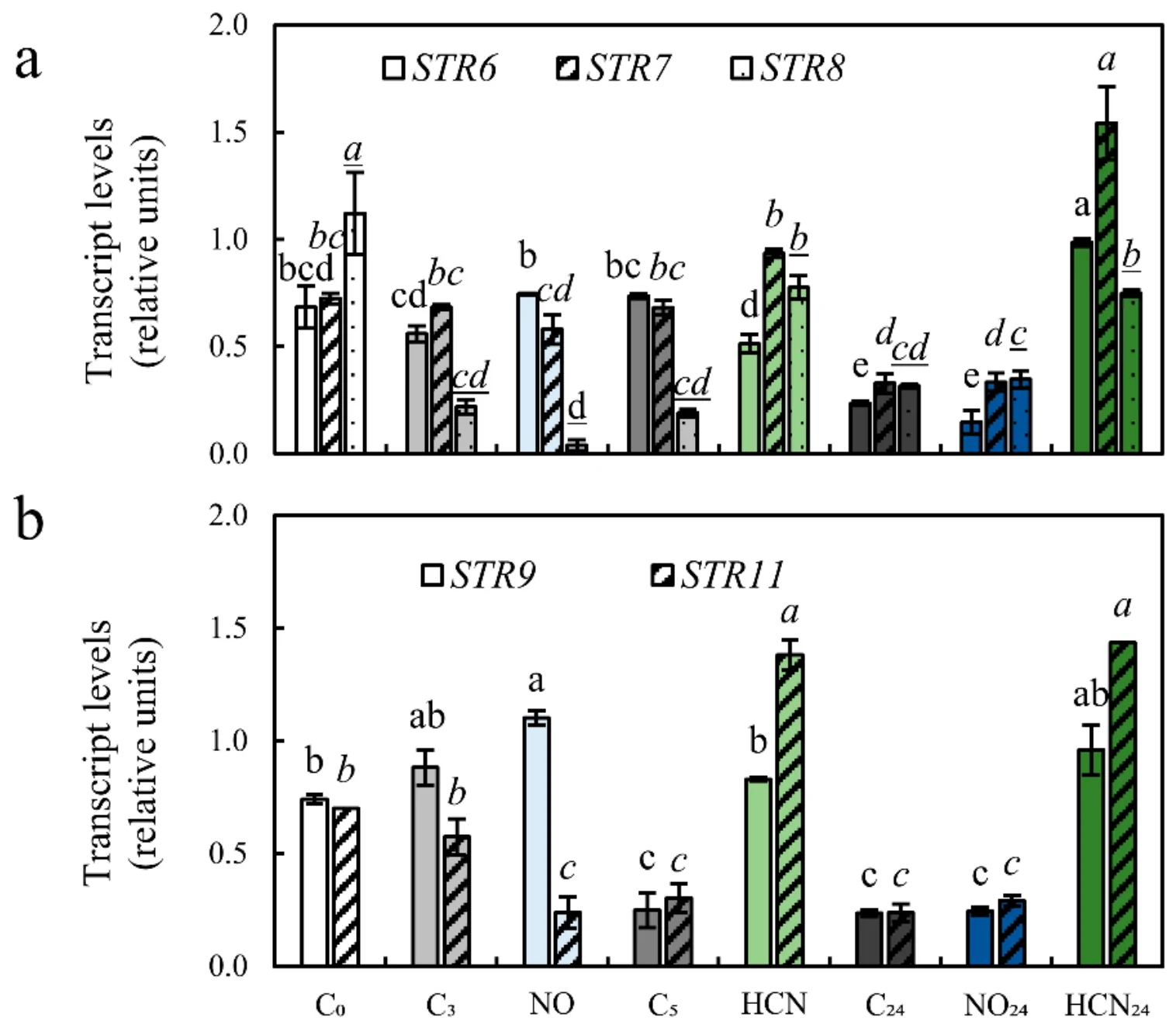
Transcript levels of (**a**) *STR6*, *STR7*, or *STR8* and (**b**) *STR9* and *STR11* in axes isolated from non-treated embryos (just after isolation, C_0_), after 3 h (C_3_), 5 h (C_5_), and 24 h (C_24_) imbibition in water and in axes of embryos just after their fumigation with NO (NO) or HCN (HCN) and after 24 h of culture after treatments (NO_24_, HCN_24_). Values are average ± SD, *n* = 3. For *STR6* transcript level, letters (a–e), *STR7* transcript level, italics (*a*–*d*), *STR8* transcript level, underline letters (*a*–*d*), *STR9* transcript level, letters (a–c) and *STR11* transcript level, italics (*a*–*c*), indicate significant differences between the experimental combinations according to Tukey’s post hoc test (*p* ≤ 0.05).

NO fumigation increased *STR6* and decreased *STR8* and *STR11* transcript levels, particularly in axes just after treatment, with no effect on other investigated genes encoding STR homologues (Figure 8a,b). In contrast, HCN exposure resulted in elevated transcript levels of *STR6*, *STR7*, and *STR8*, mainly after 24 h of culture (Figure 8a), and of *STR9* and *STR11*, just after treatment, and as culture was prolonged for 24 h (Figure 8b). Meanwhile, in embryonic axes from non-treated apple embryos, transcript levels of all analysed genes decreased as the culture was prolonged for 24 h (Figure 8a,b).

### 3.8. HCN Decreases STR12, STR14 and STR18 Transcript Levels in Apple Embryonic Axes Just After Treatment, While Increasing These Gene Transcript Levels in the Prolonged Culture

**Figure 9:**
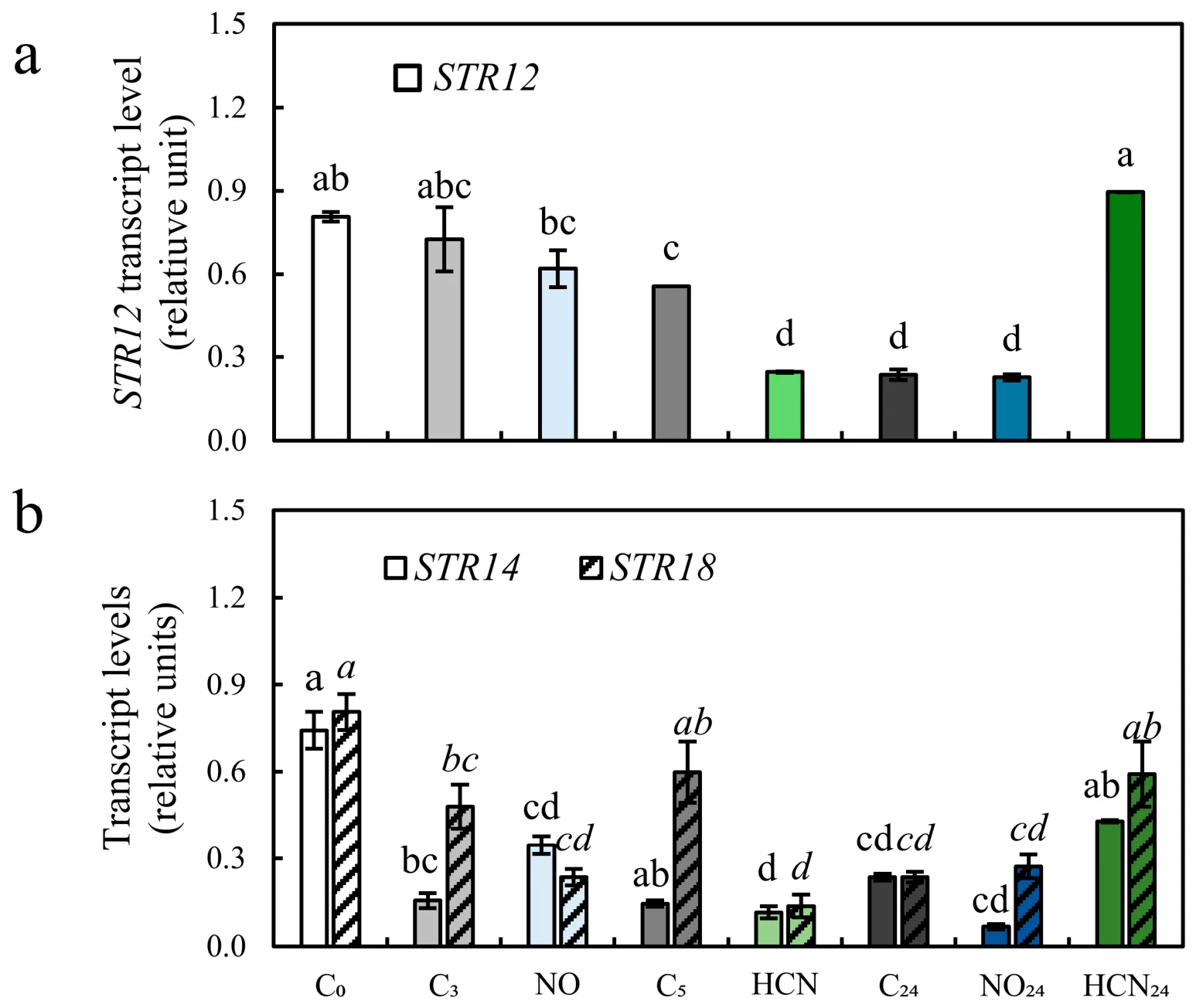
The transcript levels of (**a**) *STR12*, (**b**) *STR14* and *STR18* in embryonic axes isolated from embryos not treated (just after isolation, C_0_), after culture for: 3 h (C_3_), 5 h (C_5_) and 24 h (C_24_) and from embryos exposed to NO or HCN, and after 24 h of culture after such treatments (NO_24_, HCN_24_). Values are average ± SD, *n* = 3. For *STR12* and *STR14* transcript level, letters (a–d), and *STR11* transcript level, italics (*a*–*d*), indicate significant differences between the experimental combinations according to Tukey’s post hoc test (*p* ≤ 0.05).

HCN exposure induced an initial decrease in *STR12*, *STR14*, and *STR18* transcript levels, but its effect was reversed after 24 h of post-fumigation culture (Figure 9a,b). NO treatment did not affect the transcript levels of *STR12*, *STR14,* and *STR18* (Figure 9a,b). In control embryonic axes, *STR12*, *STR14* and *STR18* transcript levels decreased as the culture progressed (Figure 9a,b).

## 4. Discussion

Plants growing in dynamic environments encounter diverse stresses, requiring their metabolism to remain sufficiently flexible to support responses to changing conditions. This reaction depends on appropriate signalling networks that trigger short-term adjustments in enzymatic activity and long-term alterations at the transcriptomic and proteomic levels within metabolic pathways [73]. Gasotransmitters, e.g., NO or HCN, are, besides classical phytohormones, involved in such signalling [1,2,73,74]. For many years, HCN has been regarded solely as a toxic compound that has to be rapidly detoxified. However, its physiological properties resemble those of other small molecules, such as NO and H_2_S, which are toxic at high concentrations but function as important signalling agents at lower levels [75,76,77,78]. HCN can originate from exogenous sources or can be produced endogenously through multiple pathways, including those associated with ethylene, camalexin, and various cyanide-containing compounds [32]. HCN levels increase just before germination in seeds of many cyanogenic and non-cyanogenic plants [14,59]. Treatment of tomato (*Solanum lycopersicum* L.) seeds with KCN showed a pleiotropic effect on dormancy removal, which was reflected in the downregulation of seed storage proteins, thereby accelerating their degradation and stimulating glycolysis, the TCA cycle and oxidative phosphorylation [64]. In this study, we examined the impact of applied NO and HCN on β-glucosidases activity and how this might affect the pathway for HCN detoxification in apple embryos during dormancy breakage.

In our study, β-glucosidases extracted from embryonic axes of apple embryos treated with HCN or NO exhibited higher activity as measured by *p*NP released from artificial cyanogenic glycoside–*p*-NPG (Figure 2). Both HCN and NO are small gaseous molecules that can readily cross biological membranes. The moderate lipid solubility and small size of the HCN molecule [79], and the high lipophilicity of NO [80], allow them to rapidly cross membranes and enter subcellular compartments. Their permeability efficiency has not yet been experimentally compared. NO and HCN transiently affected membrane permeability (increased permeability) in apple embryos [13], which may be associated with the release of substrate (cyanogenic glycosides are stored in vacuoles) and its contact with the enzyme. High β-glucosidase activity detected after NO application may suggest increased HCN release. The stimulation of β-glucosidase activity following the application (fumigation) of apple embryos with HCN or NO points to a potent feedback regulatory mechanism. Consistently, after HCN exposure, an increased NO emission was noted from apple embryonic axes [1]. Liberation of both N-containing gasotransmitters occurs at around 14–15 days of cold stratification of apple seeds [57,60]. This implies a metabolic crosstalk between these two gases during the breakage of dormancy in apple embryos, in which one can alter the biosynthesis of the other.

In plants, two main enzymes are involved in controlling HCN level: β-CAS and STR. We observed that NO and HCN stimulated β-CAS activity (Figure 3) and increased the *CYS-C1 (β-CAS)* transcript level (Figure 4), potentially implicating this enzyme in maintaining HCN level. In the Arabidopsis cys-c1 mutant, which transiently accumulated HCN during avirulent bacterial infection, disruption of cytochrome *c* oxidase (COX)-dependent respiration, activation of AOX, promotion of ROS synthesis, and salicylic acid-mediated immune signalling were observed [81]. Additionally, analysis of T-DNA insertional mutants defective in the CYS-C1 enzyme has demonstrated that mitochondrial β-CAS activity is essential for maintaining low HCN levels, thereby supporting proper root hair development [23]. The reduction in oxygen uptake and ATP levels was observed in embryonic axes isolated from apple embryos exposed to HCN and NO [1]. Notably, elevated ROS levels were also detected after such treatments [55]. These alterations are necessary during dormancy breakage rather than during stress-response system activation in seeds, but they cannot be ruled out. Consistently, only HCN elevated the *CYS-D1* transcript level immediately after treatment (Figure 4). Cys-D1 and CYS-D2 are putative β-CAS, as was indicated in phylogenetic analyses [82], but their corresponding β-CAS activity has not been observed in vitro [41,83]. Whether elevated *CYS-D1* transcript levels after HCN lead to increased CYS-D1 translation and potent cytosolic Cys synthesis remains unknown, as CYS-D1 function is not well understood. In Arabidopsis, besides CYS-C1, CYS-D1 and CYS-D2 belonging to the β-substituted alanine synthase family, other proteins are included as three major OASTLs: OASTL-A1 (cytosolic), OASTL-B (plastidial), and OASTL-C (mitochondrial) [84] and the *S*-sulfocysteine synthase (CS26) [44]. In this study, we evaluated the transcript levels of *OASTL-A1*, *OASTL-C*, and *CS26* (Figure 5). After apple embryos were fumigated with NO, only the *OASTL-A1* and *CS26* transcript levels were elevated (Figure 5), whereas after HCN application, transcript levels of all three genes were higher, mainly after 24 h of post-fumigation culture (Figure 5). Through HCN detoxification in mitochondria by β-CAS action, co-products such as β-cyanoalanine and H_2_S are formed. H_2_S is another member of the gasotransmitter family in both animals and plants [77,85]. It inhibits oxygen consumption by binding to mitochondrial COX. In mammalian systems, both HCN and H_2_S are non-competitive inhibitors of oxygen consumption and exhibit similar inhibition constants [86]. H_2_S, like HCN, requires a proper detoxification pathway. In mitochondria, H_2_S produced as a result of HCN detoxification is metabolised by the mitochondrial isoform of OASTL(OASTL-C). This enzyme catalyses the incorporation of H_2_S to *O*-acetylserine to produce Cys (Figure 1), which, in turn, could be used by β-CAS to detoxify HCN, thus generating a cyclic pathway for HCN removal in mitochondria [87]. Increased transcript level of *OASTL-C* in apple embryonic axes after HCN fumigation may indicate a need for additional OASTL-C protein, consistent with the above-described phenomenon, to potentially regulate H_2_S and HCN level. Additionally, appropriate mitochondrial HCN metabolism in apple seeds treated with HCN is necessary to regulate COX and ATP synthesis, both of which are essential for subsequent optimal seedling development. NO did not alter *OASTL-C* transcript level (Figure 5), and this may be related to the relatively low level of H_2_S produced under such conditions, which is compromised by the optimal OASTL-C activity in seeds at that time. This suggestion requires further experimental verification. In contrast, the *OASTL-A1* transcript level increased in the apple embryonic axis after NO and HCN application (Figure 5), which may indicate a need for the production of Cys and other thiol-based compounds like glutathione. A rice (*Oryza sativa* L.) knockout mutant of *OsOASTL-A1* had significantly lower content of Cys, glutathione, and phytochelatins in roots. This suggests that OsOASTL-A1 plays an important role in non-protein thiol biosynthesis in roots [88]. Notably, the Arabidopsis *oastl-a1* mutant underwent oxidative stress due to a disruption in the balance between ROS production and scavenging [89]. After 14 days of cold stratification of apple seeds, the total glutathione content in embryonic axes increased sharply [90]. As mentioned before, around 14–15 days of this process, increases in HCN and NO emissions were observed [57,60]. These results are in agreement with the data presented in this study, indicating that both NO and HCN may be involved in the synthesis of thiol-containing molecules. Dormancy breakage of apple embryos is accompanied by overproduction of ROS, so an appropriate ROS-scavenging system is needed to maintain an optimal physiological ROS concentration. As apple seeds are of the *orthodox* type, thiol-based non-enzymatic antioxidants, rather than enzymatic ones, are involved in regulating such signalling. CS26 consistently plays an essential role in chloroplast redox control [44], although its function in seeds remains unexplored.

STR activity was stimulated by both NO and HCN in apple embryos, regardless of the culture duration (Figure 6). This suggests that NO, by stimulating β-glucosidase activity, may also activate enzymes required for HCN detoxification. Rhodanese activity has been compared in crude extracts of cyanogenic and non-cyanogenic plants, revealing no correlation between rhodanese activity and the presence of cyanogenic glycosides in individual plant species [91,92]. Moreover, in cassava (*Manihot esculenta* Crantz), a cyanogenic species, cyanogenic glucosides, SCN^−^, and rhodanese activity exhibited diurnal variation, and rhodanese activity correlated with SCN^−^ concentrations; rhodanese activity increased as the levels of cyanogenic glucosides decreased [93]. In our experiment, β-glucosidase activity may be implicated in HCN formation that potentially activates STR and β-CAS activity. Analysis of transcript levels for STR homologues (Figure 7, Figure 8 and Figure 9) is consistent with enzymatic activity occurring only after HCN application, whereas NO exposure had little effect. Although STR activity is widespread across organisms, the physiological roles of these enzymes remain incompletely defined. In mammals, STR enzymes are linked to the detoxification of cyanogenic compounds [52,94], sulphate assimilation [53], trafficking of specific sulphur compounds [95], and provision of reduced sulphur for the biosynthesis of prosthetic groups in Fe-S cluster proteins such as ferredoxin [54,96]. Despite these hypotheses, *in planta* functions of many STR isoforms remain vague. In wheat (*Triticum aestivum* L.), STR activity has been linked to resistance against the fungal pathogen *Erysiphe graminis* [97], pointing to a role in biotic stress defence. In pricklyburr (*Datura innoxia* Mill.), a cadmium-induced homologue of Arabidopsis STR9 was identified, consistent with a function in heavy-metal stress responses [98]. It has been proposed that STR proteins also contribute to redox homeostasis in distinct cellular compartments through protein–protein interactions with thioredoxins, functioning as thioredoxin-dependent peroxidases by transiently forming a sulfenate at the active-site Cys [50]. STR1 localises to mitochondria [99] and has been associated with plant senescence and HCN detoxification [100]. Ethylene and its precursor, ACC, also induce *STR1* expression [101]. The increase in transcript level of *STR1* in apple embryonic axes following HCN exposure (Figure 7a) is plausible given the proposed role of STR1 in the ethylene biosynthetic pathway, in which HCN is formed in the ratio 1:1. In apple embryos, HCN fumigation enhanced ethylene emission and lowered ACC synthesis after 4 days of the treatment. It was accompanied by a marked increase in ACC oxidase activity [102]. Thus, it was proposed that ACC can additionally undergo non-enzymatic conversion to ethylene in the presence of ROS (H_2_O_2_), HCN and NO [102]. In parallel, STR4a is unique among Arabidopsis rhodanese-like proteins in its association with chloroplast NADH dehydrogenase-like (NDH) complex, and its paralog STR4 directs the ferredoxin-NADP^+^ reductase (FNR) complex to thylakoid membranes [103], together suggesting that NDH-linked STR activity may contribute to a shared function in chloroplast electron transport. Although their roles in seeds during the early phases of dormancy breakage remain unknown, our data showing increased *STR4* and *STR4a* transcript levels after HCN exposure (Figure 7b) suggest that these proteins may participate in signalling associated with the proplastid-to-mature plastid conversion occurring later, during seedling development. Rhd domains of STR share structural similarity with the catalytic domain of Cdc25 phosphatases. In mammals, Cdc25 phosphatase dephosphorylates Cdc2 and other cyclin-dependent kinases at Thr14 and Tyr15, thereby activating the kinases and promoting cell-cycle progression [104]. Several Arabidopsis STR gene sequences-*STR4a*, *STR7*, *STR8*, *STR9*, *STR11*, and *STR14*-are annotated as members of the rhodanese/cell cycle control phosphate superfamily, but their precise role in cell-cycle regulation has not yet been experimentally defined [105]. HCN treatment of apple embryos increased *STR7*, *STR8* (Figure 8a), *STR9*, *STR11* (Figure 8b), and *STR14* (Figure 9b) transcript levels. Given that these proteins may regulate the cell cycle, HCN’s stimulation of transcription of these genes could promote cell division and support post-germination root growth, but these hypotheses need further experimental verification. Nonetheless, the high abundance and broad subcellular distribution of STR proteins in Arabidopsis and other plant species strongly suggest that these proteins fulfil multiple specialised functions, potentially including links among HCN detoxification, redox signalling, and cell-cycle control.

## 5. Conclusions

**Figure 10:**
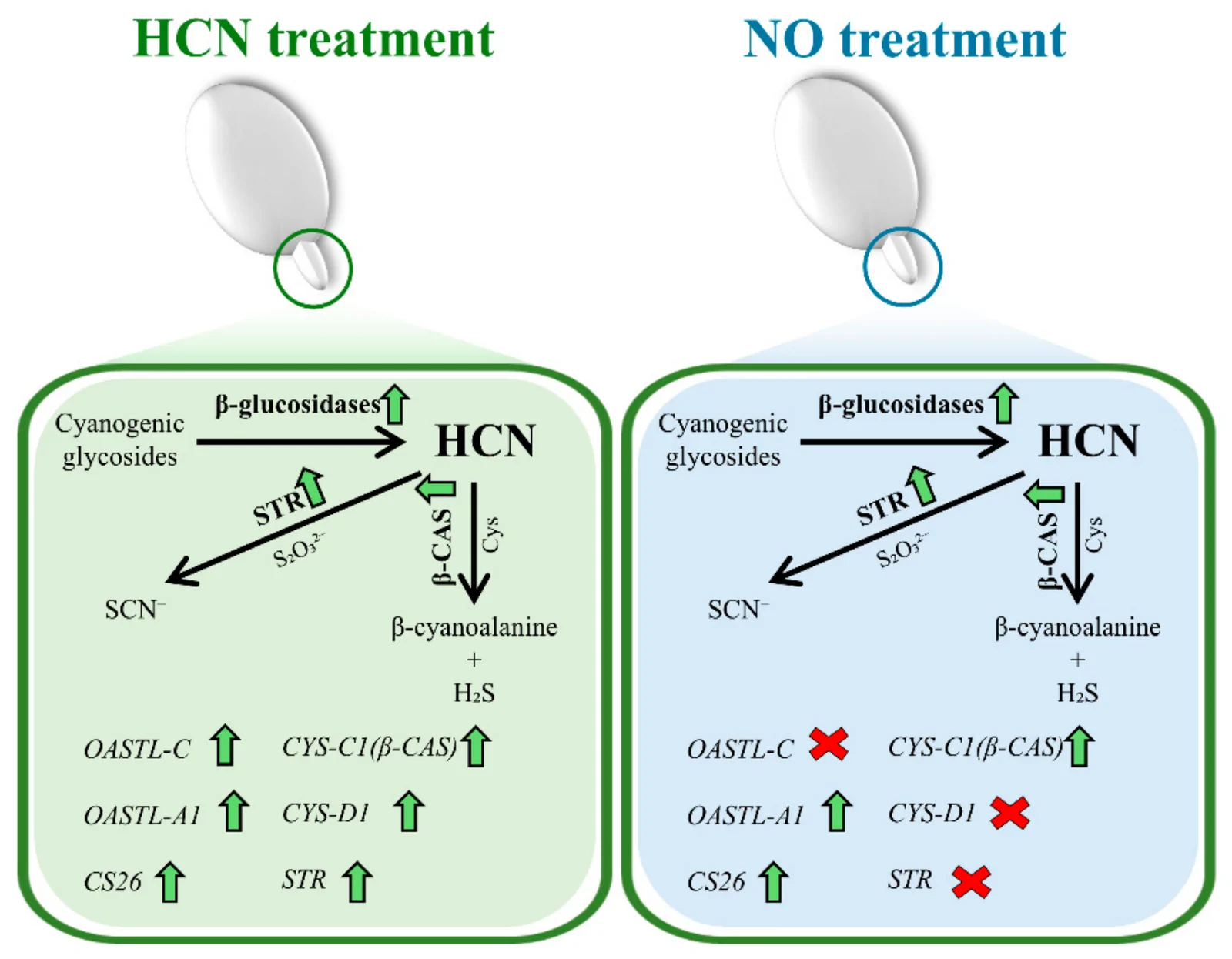
Schematic overview of the alterations in HCN metabolic pathways during NO- or HCN-induced dormancy alleviation of apple embryos. Green arrows indicate growth, and red X indicates no change.

Our study is the first one demonstrating the similarities and differences in the action of N-containing gasotransmitters (NO and HCN) concerning activities of β-glucosidases, β-CAS and STR and transcript levels of related genes in axes of apple embryos during dormancy breakage (Figure 10).

In apple embryonic axes, NO and HCN stimulate the activity of β-glucosidases (Figure 10) and increase the activity of β-CAS and STR (thiosulfate cyanide sulfurtransferase) (Figure 10), probably contributing to HCN signalling. Both gases increase *CYS-C1 (β-CAS)* and *CS26* transcript levels, yet they differentially affect the transcript levels of *CYS-D1* and genes encoding STR homologues (Figure 10). Transcriptional analyses indicate that only HCN alters the transcript level of the mitochondrial OASTL (*OASTL-C*) (Figure 10). This data, together with our previous reports [1], suggests potential metabolic crosstalk between NO and HCN, with one altering the metabolism of the other during the breakage of dormancy in apple embryos.

## Data Availability

The data presented in this study are available on request from the corresponding author.

## Supplementary Materials

The following supporting information can be downloaded at https://www.mdpi.com/article/10.3390/biology15191736/s1, Figure S1: The scheme of experimental model; Table S1: List of primers, NCBI or GDR accession numbers, amplicon sizes, and PCR amplification efficiencies; Table S2: Shapiro–Wilk test for normality for the analysed parameters.

## Abbreviations

The following abbreviations are used in this manuscript:

AOX	Alternative oxidase
β-CAS	β-Cyanoalanine synthase
COX	Cytochrome *c* oxidase
CS	Cysteine synthase
Cys	Cysteine
OASTL	*O*-acetylserine(thiol)lyase
*p*-NP	*p*-Nitrophenol
Rhd	Rhodanese domain
STR	Sulfurtransferase
