# Supplementary material for: Cyanide Metabolism in Apple Embryos During Dormancy Breakage Is Modulated by NO or HCN Application
Source: Biology (Basel). 2026 Oct 1;15(19):1736. doi: 10.3390/biology15191736 (PMC13663344; doi:10.3390/biology15191736)
Supplement: Supplementary file 1 [file biology-15-01736-s001.zip › Figure S1. The scheme of experimental model.pdf]

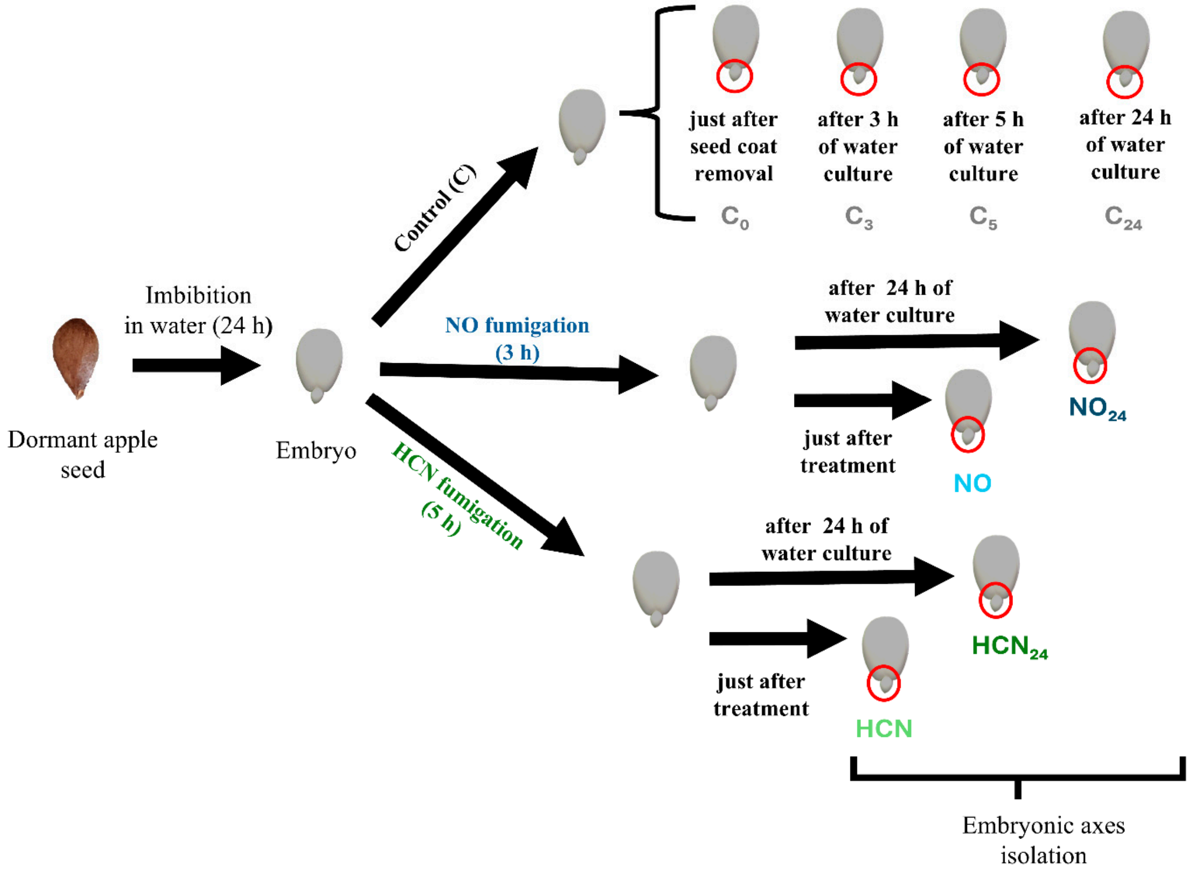

Figure S1. The scheme of experimental model. C_0 - embryos or embryonic axes isolated just after seed coat removal, C_3, C_5, C_{24} - embryos or embryonic axes isolated after 3, 5, or 24 h of water culture, HCN and NO – embryos or embryonic axes isolated just after HCN or NO treatment, NO_{24} and HCN_{24} - embryos or embryonic axes isolated after 24 h of post-fumigation culture in water.
