# Supplementary material for: Cyanide Metabolism in Apple Embryos During Dormancy Breakage Is Modulated by NO or HCN Application
Source: Biology (Basel). 2026 Oct 1;15(19):1736. doi: 10.3390/biology15191736 (PMC13663344; doi:10.3390/biology15191736)
Supplement: Supplementary file 1 [file biology-15-01736-s001.zip › Table S1. List of primers, NCBI or GDR accession numbers, amplicon sizes, and PCR amplification efficien.pdf]

Table S1. List of primers, NCBI or GDR accession numbers, amplicon sizes, and PCR amplification efficiencies.

**Gene symbol**	**Primer sequence 5'-3'**	**Encoded protein**	**Gene ID (GDR, NCBI)**	**Amplicon size (bp)**	**PCR efficiency (%)**
CYS-C1(CAS)	F: GTCAAGCAAGAGATGTTCCA<br>R: GGAGTTATCAAGCCCTTCTCT	Cysteine synthase C1; L-3-cyanoalanine synthase 1; L-3-cyanoalanine synthase 2	MD07G1135000<br>MD01G1065600	95	99.0
CYS-D1	F: TGGTGGGGATTTCATCTGGTG<br>R: GTGCAGAAGACAGATAACGCT	Cysteine synthase D1	MD04G1218500	126	103.4
OAS-A1	F: GTGGAAGGTTGTGTTGCTCG<br>R: TCTGTCCAGGTGTGATAAGG	O-acetylserine(thiol)lyase, A1 isoform	MD13G1193400<br>MD16G1194200	130	103.0
OAS-C	F: CTTCTCTGACCAATCCGCTGA<br>R: GGTTATACTGGTGGACCTTAG	O-acetylserine(thiol)lyase, C isoform	MD06G1007300	122	97.5
CS26	F: GCCTCCTCTGTCTACGCCAT<br>R: ATTTGCCACACAACCTTCGCT	O-acetylserine(thiol)lyase with *S*-sulfocysteine synthase activity, chloroplastid isoform	MD12G1013800	126	104.4
STR1	F: GATGTTGAATCTAGTGCCTCTGG<br>R: CAACTGCTTGTCCCTTGTAT	Sulfurtransferase/rhodanese with mercaptopyruvate sulfurtransferase activity (MST1)	MD10G1208500<br>MD05G1228000<br>MD05G1228700	91	97.3

STR4	F: TGAGTGGTGGAGGTGGAGAC<br>R: CTTCAGAACCTGAGACAGAA	Sulfurtransferase/rhodanese/*THYLAKOID RHODANESE-LIKE*	MD07G1147500<br>MD01G1078500	137	100.6
STR4a	F: TCCATCAAAGTGGCTGAGTT<br>R: TCCAGCCACCCTTTTGTACC	Rhodanese/Cell cycle control phosphatase superfamily protein	MD10G1147900<br>MD05G1153600	92	104.5
STR6	F: ATGTGCCTCTCTTCATTGAA<br>R: GTACAATACATGAGGACAGA	Rhodanese-like domain protein	MD04G1192000	139	104.7
STR7	F: CATGACGCTTTCCAGGTGCT<br>R: CGAACTTGTAGAAGGAGACC	Rhodanese/Cell cycle control phosphatase superfamily protein	MD01G1168900<br>MD07G1234800	115	101.9
STR8	F: ATCTGCTGTATCTATGCTGT<br>R: GATACGTGTGCCACTTCTTG	Rhodanese/Cell cycle control phosphatase superfamily protein	MD14G1244500<br>MD06G1237700	132	105.3
STR9	F: ATGTGCCTCTCTTCATTGAA<br>R: GACTCTACAAATTCAGGG	Rhodanese/Cell cycle control phosphatase superfamily protein	MD12G1061100<br>MD14G1060800	130	99.0
STR11	F: CATGTCAGAAGGGATTGAG<br>R: TCTAAACCTCCTTGAACCCA	Rhodanese/Cell cycle control phosphatase superfamily protein	MD06G1224500	91	106.0
STR12	F: ATGCAAGATCCCAGTTTCTT<br>R: CCAAATTGTCGAAGAGGAAG	Rhodanese-like domain-containing protein / PPIC-type PPIASE domain-containing protein	MD11G1184200	113	100.9

STR14	F: ATGTGAAAGAAGCTCTGAGG<br><br>R: AAGCCTGTATATTTGCACGT	Rhodanese/Cell cycle control phosphatase superfamily protein	MD07G1296000<br><br>MD01G1225500	122	103.6
STR18	F: GTCACCGTTGATGTTCAAGA<br><br>R: ATCCACATGCCCTTTCTTGT	Thiosulfate sulfurtransferase/rhodanese	MD02G1129500<br><br>MD15G1243800	99	105.1
ACT	F: CTCAGTGGTGGGTCAACTATGTTC<br>R: CTCTGGTGGAGCTACAACCTTGATC	Actin	AB638619.1	108	101.6
SAR1	F: TTGATTTGGGCGGGCATCAGATTG<br>R: TCATCAGAGAGGAGAGCATCCAGC	Small GTP-binding protein	MD04G1194800	145	104.0
