# Supplementary material for: Cyanide Metabolism in Apple Embryos During Dormancy Breakage Is Modulated by NO or HCN Application
Source: Biology (Basel). 2026 Oct 1;15(19):1736. doi: 10.3390/biology15191736 (PMC13663344; doi:10.3390/biology15191736)
Supplement: Supplementary file 1 [file biology-15-01736-s001.zip › Table S2. ShapiroWilk test for normality for the analysed parameters.pdf]

Table S2. Shapiro-Wilk test for normality for the analysed parameters.

**Experimental parameter**	**Shapiro-Wilk *p*-value**
β-glucosidase activity	0.3158
β-CAS activity	0.1888
CYS-C1(CAS)	0.2145
CYS-D1	0.4200
OAS-A1	0.2447
OAS-C	0.1001
CS26	0.5387
STR activity	0.3722
STR1	0.2305
STR4	0.3060
STR4a	0.2734
STR6	0.6937
STR7	0.9987
STR8	0.7126
STR9	0.1576
STR11	0.1454

STR12	0.0986
STR14	0.2224
STR18	0.1635
